# Prospect of genetic disorders in Saudi Arabia

**DOI:** 10.3389/fgene.2023.1243518

**Published:** 2023-09-20

**Authors:** Amerh S. Alqahtani, Raniah S. Alotibi, Taghrid Aloraini, Fahad Almsned, Yara Alassali, Ahmed Alfares, Bader Alhaddad, Mariam M. Al Eissa

**Affiliations:** ^1^ Medical Genetics Department, King Saud Medical City, Riyadh, Saudi Arabia; ^2^ Department of Clinical Laboratory Sciences, College of Applied Medical Sciences, King Saud bin Abdulaziz University for Health Sciences, King Abdullah International Medical Research Centre (KAIMRC), Riyadh, Saudi Arabia; ^3^ Division of Translational Pathology, Department of Laboratory Medicine, King Abdulaziz Medical City, Department of Genetics, King Abdullah Specialized Children Hospital, MNGHA, Riyadh, Saudi Arabia; ^4^ Division of Translational Pathology, Department of Laboratory Medicine, King Abdulaziz Medical City, Riyadh, Saudi Arabia; ^5^ Department of Genetics, King Abdullah Specialized Children Hospital, MNGHA, Riyadh, Saudi Arabia; ^6^ Research Centre, King Fahad Specialist Hospital in Dammam (KFSH-D), Dammam, Saudi Arabia; ^7^ Population Health Management, Eastern Health Cluster, Dammam, Saudi Arabia; ^8^ Research and Development Department, NovoGenomics, Riyadh, Saudi Arabia; ^9^ Medical School, AlFaisal University, Riyadh, Saudi Arabia; ^10^ Centre for Genomic Medicine, King Faisal Specialist Hospital and Research Centre, Riyadh, Saudi Arabia; ^11^ Molecular Genetics Department, King Saud Medical City, Riyadh, Saudi Arabia; ^12^ Laboratory Medicine Department, King Fahd Hospital of the University, Imam Abdulrahman Bin Faisal University, Al Khobar, Saudi Arabia; ^13^ Public Health Authority, Public Health Lab, Molecular Genetics Laboratory, Riyadh, Saudi Arabia

**Keywords:** single nucleotide variant, neurodevelopmental disorders, exome sequencing, genome sequencing, panel, diagnostic yield, Saudi Arabia

## Abstract

**Introduction:** Rare diseases (RDs) create a massive burden for governments and families because sufferers of these diseases are required to undergo long-term treatment or rehabilitation to maintain a normal life. In Saudi Arabia (SA), the prevalence of RDs is high as a result of cultural and socio-economic factors. This study, however, aims to shed light on the genetic component of the prevalence of RDs in SA.

**Methodology:** A retrospective study was conducted between September 2020 and December 2021 at King Saud Medical City, a tertiary hospital of the Ministry of Health (MOH), SA. A total of 1080 individuals with 544 potentially relevant variants were included. The index was 738, and the samples were tested in a commercialized laboratory using different molecular techniques, including next-generation sequencing.

**Result:** A total of 867 molecular genetics tests were conducted on 738 probands. These tests included 610 exome sequencing (ES) tests, four genome sequencing (GS) tests, 82 molecular panels, 106 single nucleotide polymorphism (SNP) array, four methylation studies, 58 single-gene studies and three mitochondrial genome sequencing tests. The diagnostic yield among molecular genetics studies was 41.8% in ES, 24% in panels, 12% in SNP array and 24% in single gene studies. The majority of the identified potential variants (68%) were single nucleotide variants (SNV). Other ascertained variants included frameshift (11%), deletion (10%), duplication (5%), splicing (9%), in-frame deletion (3%) and indels (1%). The rate of positive consanguinity was 56%, and the autosomal recessive accounted for 54%. We found a significant correlation between the ES detection rate and positive consanguinity. We illustrated the presence of rare treatable conditions in *DNAJC12*, *SLC19A3*, and *ALDH7A1,* and the presence of the founder effect variant in *SKIC2.* Neurodevelopmental disorders were the main phenotype for which genetics studies were required (35.7%).

**Conclusion:** This is the sixth-largest local study reporting next-generation sequencing. The results indicate the influence of consanguineous marriages on genetic disease and the burden it causes for the Kingdom of SA. This study highlights the need to enrich our society’s knowledge of genetic disorders. We recommend utilising ES as a first-tier test to establish genetic diagnosis in a highly consanguineous population.

## Introduction

Rare diseases (RDs), which are found in one in every 2000 individuals, result in life-long manifestations and cause financial and emotional burdens not only for patients but also for the families and healthcare providers of patients (WHO, 2023). RDs account for between 7,362 (OMIM, 2023) and 8,120 (Orphanet, 2023) disease cases, with about 80% of RDs being caused by genetic origin and 63% of RDS affecting children (Wright et al., 2018). The exponential advances in sequencing technology and bioinformatics interpretation have accelerated the diagnostic yield of RDs and shortened the time it takes for them to get diagnosed (Seaby et al., 2021). The prevalence of RDs in Saudi Arabia (SA) is considered high compared to other parts of the world due to the high frequency of pathogenic alleles in SA (Eissa et al., 2021; Aleissa et al., 2022). Despite efforts by the Saudi government to reduce the burden of specific diseases in the Saudi population (Saudi Ministry of Health, 2020), the prevalence of RDs is still high due to the high rate of consanguine marriages (CM) in the region (Bittles and Black, 2010). Additionally, the current screening program used in SA covers only a limited number of diseases (Saudi Ministry of Health, 2020). Several studies have investigated the genetic landscape in certain centres or regions and documented the correlations between phenotypes and genotypes (Trujillano et al., 2017; Monies et al., 2019; Bertoli-Avella, et al., 2021; Bertoli-Avella, et al., 2021). The studies reported a number of main phenotypes that are most common in the SA population; similar phenotypes have been observed mainly in metabolic diseases, neurodevelopmental disorders (NDDs), dysmorphic features, neuromuscular dystrophy and congenital malformation (Monies et al., 2019; Bertoli-Avella, et al., 2021; Bertoli-Avella, et al., 2021; Alotibi et al., 2023). In this study, we aim to recruit data from King Saud Medical City (KSMC), a tertiary hospital of the Ministry of Health (MOH), SA, with cases representing the Saudi population taken from eastern, western, northern and southern regions of SA. Herein, we report molecular genetic findings among probands referred to one of the biggest MOH genetic centres in Saudi Arabia. The data to be reported include the type of requested genetic tests, diagnostic yield, primary phenotypes, variant type, mode of inheritance and consanguinity rate. Thus, this study improves our understanding of the genetic makeup of the population of SA.

## Methodology

### Ethical approval

Approval was obtained from the ethical committee at the local Institutional Review Board at KSMC Research Centre, Ministry of Health, Riyadh, Saudi Arabia (SA), received in September 2021, for 1 year (H1R1-01-Aug21-01).

### Sample size and study design

The study was conducted retrospectively at KSMC, starting in early September 2020 and ending in late December 2021. The data was collected from patients who consented to undergo genetics testing for diagnostic purposes, and the samples were tested in a commercialized laboratory accredited by the College of American Pathologists (CAP). Several high-throughput sequencing (HTS) technologies have been performed, depending on the disease complexity and phenotype, starting with gene panel, exome sequencing (ES), or genome sequencing (GS). Furthermore, single nucleotide polymorphism (SNP) array and methylation analyses were performed. The variant classification was obtained according to the criteria of the American College of Medical Genetics and Genomics (ACMG) (Richards et al., 2015), and reported as pathogenic (P), likely pathogenic (LP), uncertain significance (VUS), benign (B), or likely benign (LB).

#### Data collection

We recruited 1080 individuals with 544 potentially relevant variants. The data were collected by medical geneticists concentrating on reporting P and LP variants initially identified during testing. Phenotypes, previous investigations, family history and consanguinity were collected. Additionally, the index cases and the genetic results of family members were documented with any incidental findings (IFs). Further assessment includes clinical evaluations, imaging, segregation analysis, laboratory testing and *In-Silico*.

### Databases

We reinvestigated all potentially relevant variants classified as P, LP and VUS for the presence of previously reported cases in VarSome and ClinVar (Kopanos et al., 2019; Landrum et al., 2016). GnomAD has been used to assess minor allele frequency (MAF)(Karczewski and Francioli, 2017). All P and LP variants were assessed if related to primary phenotype; on this basis, these variants were assigned as positive or primary and the case was considered as solved. Otherwise, it was considered inconclusive secondary if a P or LP variant was not linked to phenotype, or incidental if ACMG guidelines were followed for reporting IFs (Miller et al., 2021). The zygosity was linked to the variant and assessed to determine whether it rationalized phenotypes and the underlying disease. In certain cases, segregation analyses were performed to assess inheritance patterns.

### Statistical analysis

We included only one family member to avoid over-representation or bias of particular variations; thus (*n* = 342) were excluded from the study. Where appropriate, non-parametric chi-square tests and *post hoc* pairwise comparisons using chi-squared tests with Bonferroni adjustment were performed to assess the association of the categorical responses. All statistical analyses were performed using R Statistical Programming Language (v 4.2.2) (R Core Team, 2020) through the RVAideMemoire package (v 0.9-81-2) (https://cran.r-project.org/web/packages/RVAideMemoire/index.html). A *p*-value <0.05 was considered statistically significant.

## Results

### Demographic information and number of participants and molecular tests

**FIGURE 1:**
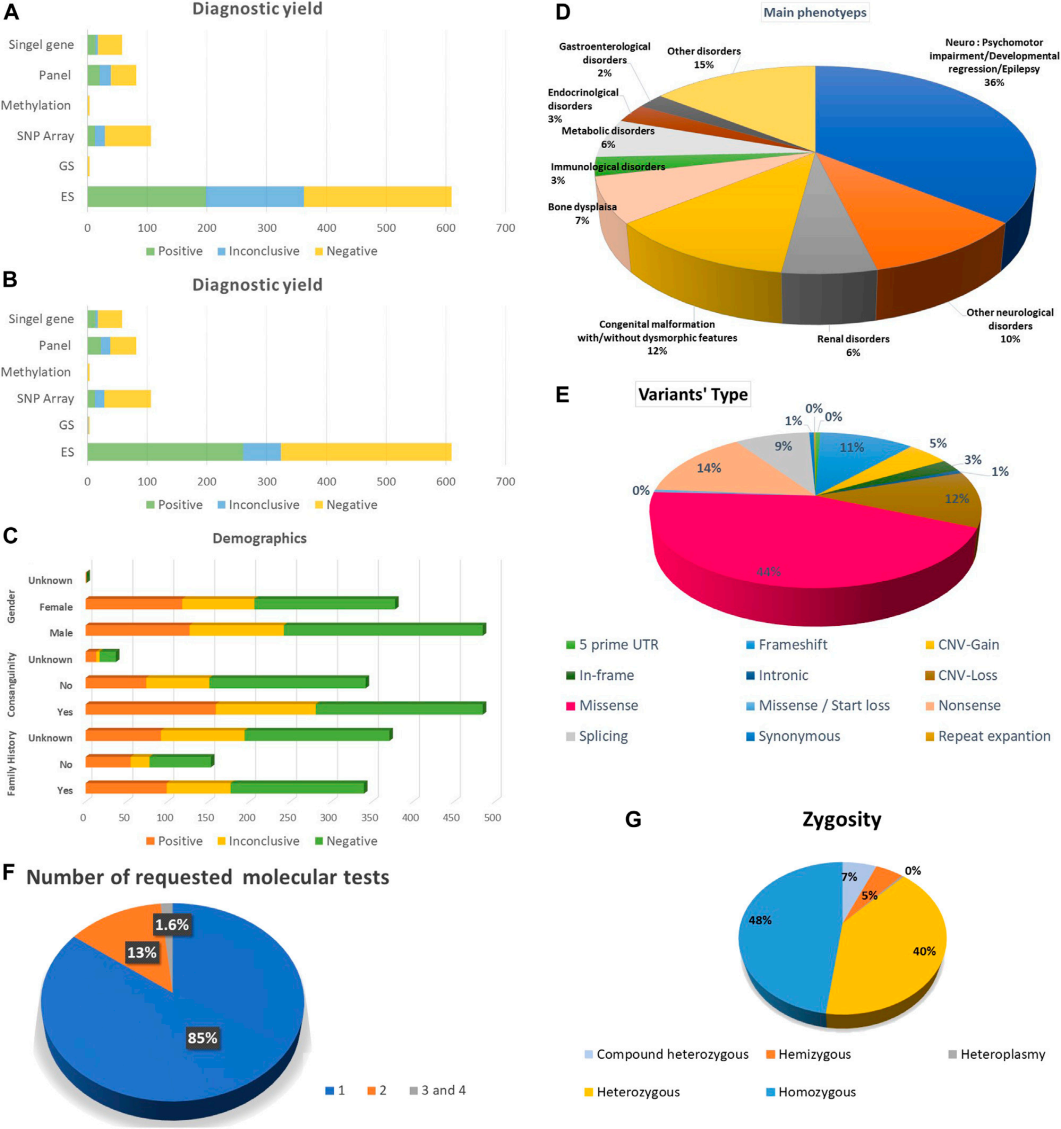
Summarization of the study’s results. **(A)** Diagnostic yield among all the requested molecular diagnostic investigations before re-classification. **(B)** Diagnostic yield among all the requested molecular diagnostic investigations after re-classification **(C)** Demographic data including gender, family history and consanguinity. **(D)** The main phenotypes for the index cases. **(E)** Variant types. **(F)** Frequency of requested molecular tests per individual. **(G)** Zygosity of the potentially encountered variants.

The number of tested probands was 738, belonging to 738 families. Probands underwent (867) molecular genetics tests, including (610) ES (598) Solo, four Duo and eight as Trio or quad, four GS, (82) several molecular panels, (106) SNP array, (4) a methylation study, (58) a single-gene study and three mitochondrial genome sequencing tests. 627 (85%) probands performed one molecular test, 99 (13%) had two molecular tests, and 12 (1.6%) underwent three and four molecular investigations. Of those who performed genetic tests in this study, 56% were males, and 43% were females. The positive consanguinity status in our study was 56%. The presence of known family history (FH) was 39.3%, while no FH was 17.7% and unknown FH status was 42.9%. Demographic data is available in Figure 1.

### Diagnostic yield

The diagnostic yield of molecular genetics studies was ES 198 (32%), and after reanalysis of inconclusive variants, the diagnostic yield increased to 255 variants (41.8%). The upgraded data was published in another article. Among the ES trio, three demonstrated the presence of positive results and one inconclusive result. Furthermore, we have one extended family member, ES, with a positive result. In addition, there is one ES duo with a positive result and another one with an inconclusive result. The detection rate of panels is 20 (24%). After reanalysis of inconclusive variants, the detection rate was augmented to 22 (26%). The detection rate was 13 (12%) for SNP array, 14 (24%) for single gene, one (25%) for methylation study and zero for GS and mitochondrial genome. Detailed information about the diagnostic yield of all molecular tests is provided in (Figure 1).

### Types of variants

Approximately 544 potential variants were identified. Single nucleotide variants (SNV) accounted for the majority of our variants 371 (68%), among them 246 (45%) missense, 78 (14%) nonsense and 3 (1%) synonym variants. Other ascertained variants included frameshift 61 (11%), deletion 55 (10%), duplication 29 (5%), splicing 48 (9%), in-frame deletion 14 (3%), indels 5 (1%) and one repeat expansion in *FMR1* (Figure 1). In addition, structural variants were detected as copy number variant (CNV) 84 (16%), one derivative of unbalanced translocation, one tetrasomy 18p and one trisomy 20p. Six aneuploidies, including Trisomy 21, Turner syndrome, Klinefelter syndrome, one mosaic Trisomy 21 and Klinefelter syndrome, were recognized**.**

### Zygosity and mendelian inheritance

Half of the ascertained variants followed the autosomal recessive (AR) mode of inheritance (54%), which belonged to homozygous variants 239 (48%) and compound heterozygous 32 (6%). We also observed X-linked disorders in males, with 26 (5%) hemizygous. Finally, we observed 200 individuals account for (40%) as heterozygous autosomal dominant (AD) or X-Linked dominant in females (Figure 1).

### IFs and double mutations

We had 19 double mutations: AR and AD 5 (24%), X-linked/AR 4 (19%), AR/AR or AD/AD 3 (14%) and X-Linked/AD 2 (10%). The majority of dual diagnoses were explored by ES (17). The reported IFs were 15 (2%); however, they are underrepresented in our probands because several probands’ families refused to receive information in this regard.

### Frequently observed phenotypes in probands

The main phenotypes for which molecular genetics investigations were requested were psychomotor impairment, developmental regression and epilepsy 309 (35.7%); other neurological disorders were 90 (10.4%). Congenital malformation with or without dysmorphic features was 104 (12%), bone dysplasia was 62 (7.1%), and renal disorders and metabolic disorders were 52 (6%). Endocrinological and Immunological disorders were 25 (2.9%) and 26 (3%), respectively. The other disorders were 125 (14.6%) (Figure 1).

### Frequently observed pathological or likely pathological variants

Haematological disorders have been frequently noticed as secondary findings, including *G6PD* (NM_000402.3):c.653C>T (OMIM 300908), *HBB* (OMIM:613985) and (OMIM: 603903). *SLC26A3* (NM_000111.2):c.559G>T (OMIM: 214700), *CA2*(NM_000067.2):c.232 + 1G>A (OMIM: 259730), *FGFR3*(NM_001163213.1):c.1144G>A (OMIM: 100800), *PKHD1*(NM_138694.3):c.4870C>T causing (OMIM: 263200), *SKIC2* (NM_006929.4):c.3561_3581del (OMIM: 614602), *SMN1* (exon 7 and 8 deletions) (OMIM: 253300, 253,550, 253,400, 271,150), *BRCA1*(NM_007300.3):c.4136_4137del causing (OMIM: 604370) as IFs (Supplementary Table S2).

The list of P and LP variants for SNV, INDELS, small deletion, small supplication and repeat expansions and the detailed phenotypes are available in Supplementary Table S1.

### Statistical analysis

A chi-square test of independence was conducted for each diagnostic modality to see if there was a link between the test outcome and consanguinity, family history, or the number of phenotypes. This was followed by *post hoc* pairwise comparisons using chi-squared tests with Bonferroni adjustment if significant. No significant associations were found for ES between the test outcome and family history or number of phenotypes (*p* = .231, .265, respectively). However, a significant relationship was found between the test outcome and consanguinity (*p* = .001), and a *post hoc* test showed an increase in consanguinity for positive tests (O = 132 vs. E = 99, *p* = .001). No significant associations were found for the array between the test outcome and consanguinity (*p* = .428), but a significant relationship with a family history or the number of phenotypes was found (.014, 032, respectively). No significant associations were found for the single gene between the test outcome and consanguinity, family history, or the number of phenotypes (*p* = .426, .771, .197, respectively). No significant associations were found for the panel cases between the test outcome and consanguinity, family history, or the number of phenotypes (*p* = 0.434, 0.576, 0.175, respectively). Detailed statistical tests are provided in Supplementary Tables S3A–Q.

## Discussion

Few large-scale genomic analysis studies in the SA context have been published. The data in these studies have been obtained retrospectively from samples used for diagnostic purposes (Monies et al., 2019; Alfares et al., 2020). Therefore, to help compensate for this knowledge gap, we wanted to share our centre’s experience. The initial diagnostic yield of ES in this study was 32%; however, the extensive reanalysis of variants led to upgrading of potential causative variants to approximately (57) P and LP, thus enhancing the diagnostic rate of ES to 41.8%, a number close to national observations (43% and 49%) (Monies et al., 2017; 2019).

Trio and quadrigal Exome analyses were performed on eight subjects from different families, so we cannot prove the efficiency of ES trio over ES solo in terms of improving diagnostic yield. However, concerning local data, performing ES/GS as Trio or extended family-based analysis in comparison to ES alone led to no significant benefit with regard to ameliorating the ES detection rate. The main benefit of the ES trio was facilitating the downstream analysis by excluding candidate variants and reducing the need for segregation analysis (Alfares et al., 2020). This could be because trio analysis might be more beneficial in the (*de novo*) AD pattern of inheritance than in AR disorders (Clark et al., 2018). No significant advantage of utilising GS over ES was established, which could be because GS was requested for complex unsolved cases using ES and array-based technology (Alfares et al., 2018). In addition, to date, the interpretation of GS’s intronic regions remains challenging in diagnostic settings, and most known variants of causative disorders are within the codon region (Stranneheim et al., 2021)**.**

In our recruited data, the CM rate was high (56%); thus, homozygous AR variants accounted for nearly 50% of the total variants, which is similar to the finding of previous local publications (Alfares et al., 2017). We demonstrated a significant correlation between positive ES results and CM, which is in agreement with previous observations (Al-Dewik et al., 2019). The detection yield of ES in the outbred population is less than 30%, with AD disorders (*de novo*) being predominant, accounting for more than half of positive cases, followed by AR disorders as compound heterozygous (Lee et al., 2014; Yang et al., 2014). Therefore, a high consanguinity rate is vital to improving ES’s diagnostic detection rate.

Neurological-related phenotypes, including psychomotor retardation, ID and seizures, are the main phenotypes encountered for which genetic studies are requested (35%–45%), followed by congenital malformations (23.5%); these phenotypes have also been identified previously in several publications on the SA population (Retterer et al., 2016; Monies et al., 2017). This could be because the vast majority of the diagnostic genetic tests are performed in paediatrics (Bertoli-Avella et al., 2021). Of note, the diagnostic rate of ES for neurodevelopmental disorders is 36% in the outbred group and 46% in the consanguine population; for congenital malformations, the rate is around 36% (Yang et al., 2014; Al-Dewik et al., 2019). Thus, ES should be the first-tier genetic test for NDD.

The rate of double mutations by ES was 2.4% in our cohort, 6% in the local study and less than 1% in the outbred population. The higher double mutations rate in our community might be attributable to the high CM rate in the Saudi community (Retterer et al., 2016; Alfares et al., 2017).

Based on data from two large-scale studies in the Arabian peninsula, the IFs following the ACMG guidelines have been increasing in recent years (1.5%–3.9%) (Monies et al., 2017; Al-Dewik et al., 2019). The rate of IFs among ES in the Saudi study continues to rise, and is currently at 48 variants (8%). This might be explained by considering the IF rate from non-index data used in ES-Trio and extended family member analysis, which represents 54% of the IF rate, and the fact that nine novel IF variants have been reported (Aloraini et al., 2022). In addition, knowledge of IF variants causing the disease in SA has been improving in recent years due to the establishment of an in-house database and accumulation of knowledge from prior publications. Of note, ACMG’s list of recommended IFs has been expanding. Currently, it contains 78 genes, but as this number increases, the percentage of IFs may be expected to rise in ES and GS studies in the coming years (Miller et al., 2022).

Monies et al. (2019) attributed founder effect variants to P and LP variants in SA. Two out of nine variants were observed three times in our study. The first one observed was *SKIC2* (NM_006929.4):c.3561_3581del; p. (Ser1189_Leu1195del), which is causative for Trichohepatoenteric syndrome type 2 (OMIM: 614602). To the best of our knowledge, this is the first time this variant has been observed in a Saudi study. This observation is expected to enhance our knowledge regarding the founder effect variant in SA. The other one is *PKHD1*(NM_138694.3):c.4870C>T; p. (Arg1624Trp), responsible for polycystic kidney disease type 4 with or without polycystic liver disease (OMIM: 263200). This variant has seldom been observed in SA and other countries; thus, we could not consider this variant as a founder effect (Obeidova et al., 2015; Edrees et al., 2016; Al Alawi et al., 2020). However, to confirm the presence of founder effect mutation, we need a larger number of cases within the various regions of SA.

Furthermore, we observed rare treatable metabolic disorders or NDD-related variants in our study. A biallelic pathogenic variant in *DNAJC12* (NM_021800.2):c.214C>T (p.Arg72*) that is responsible for AR mild non-BH4-deficient hyperphenylalaninemia (OMIM: 617384) was identified in a patient exhibiting hyperphenylalaninemia (HPA). This gene prevents misfolding of phenylalanine hydroxylase (PAH), tyrosine hydroxylase and tryptophan hydroxylase (Straniero et al., 2017). The aforementioned variant has recently been reported in three probands manifesting HPA and mild neurological symptoms and who were responsive to a tetrahydrobiopterin (HB4) loading test (van Spronsen et al., 2018). Besides HPA, this gene has been linked to variable degrees of neurological phenotypes, including cognitive impairment, dystonia, early onset parkinsonism and the presence of abnormality of brain neurotransmitters (Anikster et al., 2017). The aforementioned disorder responds to protein diet restriction, early intervention of HB4 therapy and, possibly, to neurotransmitter precursors L-DOPA/carbidopa and 5-hydroxytryptophan (Anikster et al., 2017). We would like to emphasise that *DNAJC12* should be considered as a differential diagnosis for HPA after excluding PAH deficiency, phenylketonuria and HB4 disorder metabolism. Furthermore, we identified biotin responsiveness basal ganglia disease (OMIM: 607483) due to a pathogenic, homozygous variant in *SLC19A3* (NM_025243.3):c.1264A>G, p. (Thr422Ala). This proband exhibited episodic encephalopathy, ataxia, spastic paraparesis, confusion, irritability, dysarthria, dysphagia and bilateral ptosis, and bilateral basal ganglia lesions. This variant has been observed in the Saudi population, and the aforementioned disorder is known to respond to early intervention with high-dose thiamine and biotin (Algahtani et al., 2017). Additionally, we illustrated the presence of pyridoxine-dependent epilepsy (OMIM: 266100) in a patient manifesting neonatal seizure who had a sibling presenting a similar manifestation. This proband has a pathogenic variant at homozygous status in the antiquitin gene, *ALDH7A1* (NM_001182.3):c.1279G>C; p. (Glu427Gln). This gene plays an essential role in the lysine degradation pathway; thus, the presence of a pathogenic variant causes accumulation of alpha-amino adipic semialdehyde (AASA), leading to accumulation of piperideine-6-carboxylate (P6C), causing the inactivation of pyridoxal 5-prime phosphate (PLP), which is the active form of pyridoxine (Pena et al., 2017). Patients affected by the above-mentioned disorder respond to large daily supplements of vitamin B6, pyridoxine (Proudfoot et al., 2013).

Performing several molecular diagnostic investigations for unsolved cases lengthens the diagnostic odyssey (an average of 5 years), thereby increasing the average cost of genetics diagnostic investigations per individual (Sawyer et al., 2016; Smith et al., 2019; Bertoli-Avella et al., 2021). Monies et al. (2019) demonstrated that 78% of ES cases did not have a precise initial clinical diagnosis by requesting physicians. Furthermore, genetic heterogenicity, atypical presentation of some genetic disorders and an absence of local guidelines for the diagnostic approach for paediatric genetic diseases might encourage a habit of requesting several genetics tests prior to reaching final diagnosis and therefore lengthen the diagnostic odyssey (Armour et al., 2016; Sawyer et al., 2016; Clark et al., 2018; Smith et al., 2019). The limitation of our study is that it represents the experience of one testing centre, and that the vast majority of the age group is paediatric; hence, adult and prenatal experiences might be underrepresented. The study is also limited by the current state of knowledge with regard to disease-causing variants in our region.

In conclusion, this massive study is the sixth-largest national study reporting molecular findings of next-generation sequencing-based technology in SA. We are sharing our experience to enhance the understanding of variants that are causative of rare genetic disorders in our community, for which there is thus far no national genetic disorder database for reporting disorder-causative variants. We illustrated that inherited genetic disorders in the Saudi population followed the AR mode of inheritance (homozygous variant). We helped refine the state of knowledge regarding the founder effect variant in SA. We showed the power of ES in detecting treatable conditions. Considering the high diagnostic rate of ES among other molecular genetics modalities in highly consanguineous regions, ES as a solo-based analysis is recommended as the first-tier genetics tool in the paediatric age group.

## Data Availability

The original contributions presented in the study are publicly available. This data can be found here: https://www.ncbi.nlm.nih.gov/clinvar/, accession numbers SCV003927798, SCV003927973.
